# Meta-analysis of Maternal and Fetal Outcomes of Crimean-Congo Hemorrhagic Fever during Pregnancy

**DOI:** 10.3201/eid3210.260383

**Published:** 2026-10

**Authors:** Bahar Donk, Deniz Güllü, Ahmet Mirhan Cırık, Handan Alay, Faruk Karakeçili, Kemalettin Özden, Fatma Kesmez Can, Orçun Barkay, Natalia Pshenichnaya, Önder Ergönül

**Affiliations:** Graduate School of Health Sciences, Koç University, Istanbul, Turkey (B. Donk, D. Güllü); School of Medicine, Koç University, Istanbul (B. Donk, D. Güllü, Ö. Ergönül); Koç University—İşBank Center for Infectious Diseases (KUISCID), Istanbul (B. Donk, D. Güllü, Ö. Ergönül); School of Medicine, Marmara University, Istanbul (A.M. Cırık); School of Medicine, Atatürk University, Erzurum, Turkey (H. Alay, K. Özden, F.K. Can); School of Medicine, Binali Yıldırım University, Erzincan, Turkey (F. Karakeçili, O. Barkay); Central Research Institute of Epidemiology, Moscow, Russia (N. Pshenichnaya); Russian Medical Academy of Continuous Professional Education, Moscow, Russia (Ö. Ergönül)

**Keywords:** Crimean-Congo hemorrhagic fever, viruses, CCHF, vector-borne infections, zoonoses, pregnancy, maternal mortality, fetal loss, ribavirin, meta-analysis

## Abstract

Crimean-Congo hemorrhagic fever (CCHF) during pregnancy is associated with severe maternal and fetal complications, but evidence remains limited to heterogeneous case reports. We synthesized patient-level data from published and unpublished cases identified through systematic database searches and direct collaboration with centers in CCHF-endemic areas. We harmonized patient-level clinical, laboratory, obstetric, treatment, and outcome data for 55 pregnancies. The maternal mortality rate was 28.8% (15/52), and fetal loss occurred in 57.4% (31/54) of cases. Maternal mortality rates increased with advancing gestation, reaching 31.3% in the third trimester, but fetal loss was highest (85.7%) in the first trimester. Ribavirin treatment during the third trimester was associated with lower observed maternal mortality rates (odds ratio 0.33, 95% CI 0.13–0.84; p = 0.031). Our findings highlight gestational age–specific risks of CCHF and support the need for standardized management protocols and strengthened CCHF surveillance during pregnancy in endemic regions.

Crimean-Congo hemorrhagic fever (CCHF) is a life-threatening tickborne disease caused by CCHF virus (CCHFV) (*1*). CCHF is endemic across Africa, Asia, Eastern Europe, and the Middle East and is expanding geographically toward historically nonendemic regions, including Western and Southern Europe (*2*–*5*). Transmission occurs primarily through bites of infected *Hyalomma* spp. ticks but also through contact with the blood or tissues of viremic livestock or, less commonly, through healthcare-related exposure (*6*). Clinical manifestations typically appear after a short incubation period and begin with a nonspecific febrile prodrome, which progresses to hemorrhagic manifestations and multiorgan system failure in some patients (*1*). Reported case-fatality rates vary from 5% to 40% depending on the setting, outbreak dynamics, and access to supportive care (*7*).

Pregnancy represents a uniquely vulnerable physiologic state in the context of viral hemorrhagic fevers because of pregnancy-associated immunologic modulation, altered coagulation pathways, and the fetoplacental unit (*8*,*9*). Although data remain limited, CCHF during pregnancy has consistently been associated with disproportionately high maternal and fetal mortality rates, as well as severe obstetric complications including massive hemorrhage, disseminated intravascular coagulation, spontaneous abortion, intrauterine fetal demise, and preterm delivery (*10*,*11*). Vertical CCHFV transmission has also been reported, raising additional concerns regarding neonatal outcomes and postnatal transmission risk (*12*).

Despite those severe outcomes, evidence-based management of CCHF during pregnancy is not well-established. Most published data consist of isolated case reports or small case series, often lacking detailed clinical timelines or consistent reporting of maternal, fetal, and neonatal outcomes. As a result, key clinical questions, including predictors of maternal death, determinants of fetal loss, and the potential role of antiviral therapy, remain unclear. In addition, pregnant persons are routinely excluded from clinical trials, including those evaluating antiviral agents such as ribavirin, further limiting evidence-based guidance in this population.

We conducted a meta-analysis of published cases of CCHF during pregnancy and integrated newly identified, previously unpublished cases from endemic regions. By combining detailed clinical, laboratory, obstetric, and outcome data at the patient level, we aimed to characterize maternal, fetal, and neonatal outcomes; identify clinical and laboratory predictors of adverse outcomes; and provide comprehensive evidence to inform clinical management and risk stratification in this neglected but high-risk population.

## Methods

### Study Design and Reporting

We designed a study to synthesize patient-level data from published and unpublished cases of CCHF during pregnancy. We conducted and reported this study in accordance with PRISMA-IPD guidelines (*13*). We did not prospectively register a formal study protocol and did not register the review in PROSPERO or any other registry.

### Search Strategy

We searched for reported CCHF cases published from database inception through October 31, 2025, from the PubMed Ovid MEDLINE, Scopus, Web of Science, and Cochrane Library databases. We conducted the search on October 31, 2025, by using keywords related to pregnancy and CCHF (Appendix 1). We imported all records identified through the database search into EndNote for macOS (Clarivate Analytics) and removed duplicates. We performed screening and full-text assessment for studies published in English and manually screened reference lists in eligible studies to identify additional relevant articles.

### Inclusion and Exclusion Criteria

We included studies that described >1 pregnant patient who had clinically confirmed or laboratory-confirmed CCHF and that included individual patient-level clinical or outcome data. Study types included case reports, case series, observational studies, and review articles. We excluded review articles without primary patient-level data. We retrieved full-text articles meeting the inclusion criteria and assessed articles in detail. In addition to published studies, we incorporated unpublished cases that met the inclusion criteria.

### Study Selection

Two independent reviewers (B.D. and A.M.C.) screened titles and abstracts for eligibility. We then assessed full-text articles by using predefined inclusion criteria. We resolved disagreements through consultation with a third reviewer (D.G.).

### Case Identification and Data Collection

Two reviewers (B.D. and A.M.C.) independently extracted patient data from included studies by using a standardized prepared form in Excel (Microsoft). For unpublished cases, we put out a call through the CCHF Consortium in Turkey. Participating centers provided eligible cases by using a prepared Excel data collection sheet. In addition, we directly contacted 1 collaborator in Russia to identify potential eligible cases. We reviewed all contributed cases for completeness, internal consistency, and adherence to study inclusion criteria.

### Variables and Outcomes

Extracted variables included maternal demographic characteristics, exposure history, diagnostic methods, clinical manifestations, laboratory parameters at admission, gestational age at infection, treatment, and maternal and fetal outcomes. The primary maternal outcome was maternal death. The primary fetal outcome was fetal loss, including spontaneous abortion, intrauterine fetal demise, and neonatal death. Secondary analyses evaluated trimester-specific outcomes and explored associations between ribavirin exposure and maternal death.

### Missing Data 

We did not impute missing data. We performed all analyses by using available data only and explicitly reported the denominator for each analysis to reflect the number of cases with complete information for the relevant variable.

### Risk of Bias Assessments

Because most included studies were case reports or small case series without comparator groups, formal risk of bias tools were not applicable. Potential sources of bias included selective reporting and incomplete clinical data. We considered those limitations in the interpretation of findings.

### Statistical Analyses

We used SPSS Statistics 31.0.1 for macOS (IBM Corp.) for all statistical analyses. We summarized continuous variables as median (IQR) because of nonnormal distribution and limited sample size. We presented categorical variables as frequency (%). We conducted maternal mortality analyses using χ^2^ test for categorical comparisons and applied Fisher exact test when expected frequency in any cell was <5. We used univariate logistic regression analysis to assess associations between ribavirin use and maternal death. We reported results as odds ratios (ORs) with 95% CIs and corresponding p values. All tests were 2-sided, and we considered p<0.05 statistically significant. We used R version 4.5.2 (The R Project for Statistical Computing) to generate geographic distribution maps and related figures. To assess the robustness of the findings, we performed a sensitivity analysis that included only laboratory-confirmed cases determined by PCR, ELISA, or both.

## Results

### Study Selection

The database search identified a total of 274 articles. After removing duplicates, 173 unique records remained, from which 19 articles met our inclusion criteria. We identified 2 additional articles, 1 from a journal published in Turkey and 1 located through reference screening but not found in our initial database search. We also incorporated 1 unpublished case from Russia and 7 newly identified cases from Turkey, resulting in a total of 55 included cases (Figure 1) (Appendix 2). We did not identify any major discrepancies or data integrity concerns.

**Figure 1:**
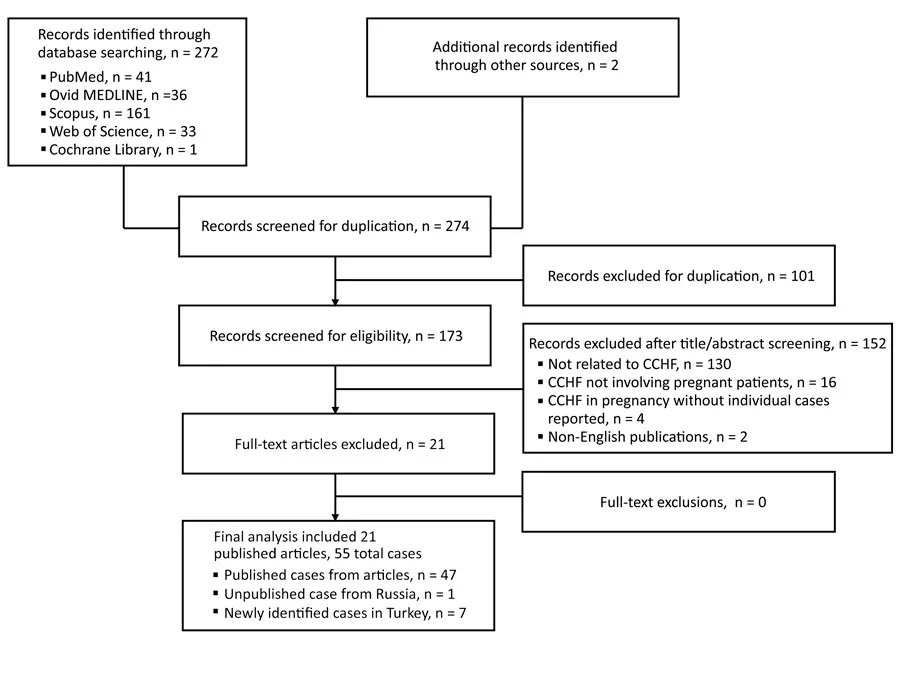
Flowchart of literature search and study inclusion for meta-analysis of maternal and fetal outcomes of CCHF during pregnancy. CCHF, Crimean-Congo hemorrhagic fever.

### Demographic Characteristics

All reported cases were described from Eastern Europe, the Middle East, and Africa. Turkey had the most (43.6%, 24/55) reported cases, followed by Iran (18.2%, 10/55) and Russia (14.5%, 8/55) (Figure 2). Maternal age was reported for 37 cases; median age was 26 (IQR 20.5–30) years. Gestational age at infection was reported for 40 cases; median gestational age was 21 (IQR 16–34) weeks, distributed across the first (n = 7), second (n = 16), and third (n = 17) trimesters. Tick exposure was reported in 20/30 (66.7%) women. Laboratory-confirmed diagnosis was available for 72.7% (40/55) of cases (Table 1).

**Figure 2:**
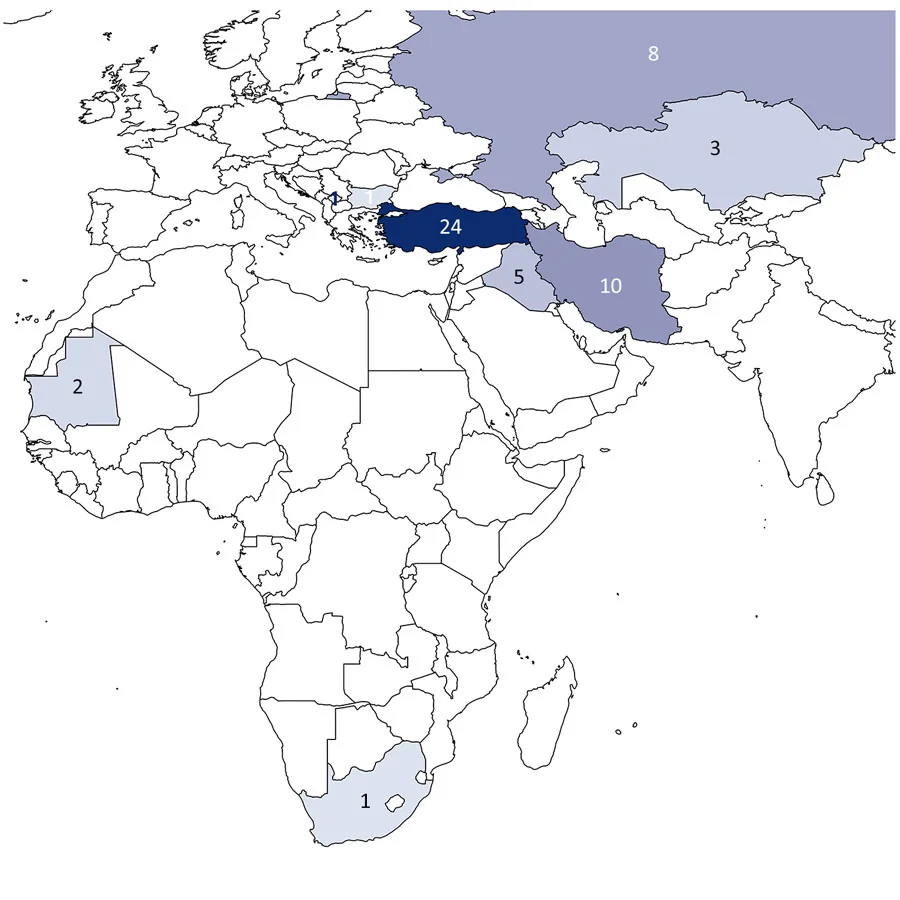
Geographic distribution of cases identified from a meta-analysis of maternal and fetal outcomes of Crimean-Congo hemorrhagic fever during pregnancy. Countries are shaded according to the number of cases; case counts are labeled on each country. Map generated by using R version 4.5.2 (The R Project for Statistical Computing).

**Table 1:** Characteristics of patients from a meta-analysis of maternal and fetal outcomes of Crimean-Congo hemorrhagic fever during pregnancy*

Characteristics	Value
Reported country, n = 55	
Turkey	24 (43.6)
Iran	10 (18.2)
Russia	8 (14.5)
Iraq	5 (9.1)
Kazakhstan	3 (5.4)
Mauritania	2 (3.6)
South Africa	1 (1.8)
Bulgaria	1 (1.8)
Kosovo	1 (1.8)
Tick bite history, n = 30	
Y	20 (66.7)
N	10 (33.3)
Median (IQR) maternal age at infection, y; n = 37	26 (20.5–30)
Median (IQR) gestational age at infection, wks; n = 40	21 (16–34)
Trimester at infection, n = 40	
First	7 (17.5)
Second	16 (40.0)
Third	17 (42.5)
Clinical signs and symptoms at initial admission, n = 55	
Fever	41 (74.5)
Myalgia	21 (38.2)
Abdominal pain	4 (7.3)
Nausea and vomiting	11 (20.0)
Headache	15 (27.3)
Rash	10 (18.2)
Malaise	9 (16.4)
Chills	11 (20.0)
Loss of appetite	8 (14.5)
Somnolence	1 (1.8)
Vaginal bleeding	13 (23.6)
Mucosal hemorrhage other than vaginal bleeding	22 (40.0)
Free fluid on abdominal ultrasonography	2 (3.6)
Diagnostic method, n = 47	
PCR	20 (42.6)
ELISA	3 (6.4)
PCR and ELISA	17 (36.2)
Clinical	3 (6.4)
Autopsy	3 (6.4)
Maternal death by trimester, n = 52	15 (28.8)
First	0
Second	3 (18.8)
Third	5 (31.3)
Unknown	7 (53.8)
Fetal death by trimester, n = 54	31 (57.4)
First, n = 7	5 (71.4)
Second, n = 16	5 (31.3)
Third, n = 17	9 (52.9)
Unknown, n =14	12 (85.7)
Concurrent maternal and fetal death, n = 33	11 (33.3)
Median (IQR) gestational age at delivery, wks; n = 19	38 (37–40)
CCHF status of fetus, no.	
IgM-negative	4
IgM-positive	1
PCR-positive	4
PCR-negative	3

*Values are no. (%) except as indicated. CCHF, Crimean-Congo hemorrhagic fever; IQR, interquartile range.

### Clinical Manifestations and Initial Laboratory Results

Fever (74.5%), mucosal bleeding (40%), myalgia (38.2%), headache (27.3%), and vaginal bleeding (23.6%) were the most frequently reported signs and symptoms (Table 1). Laboratory evaluations demonstrated marked cytopenia and coagulation abnormalities and median values were as follows: platelet count (n = 35 patients) 58 × 10^3^/µL (IQR 24–115 ×10^3^), leukocytes (n = 34) 3,700 cells/mL (IQR 2,825–5,075/mL), aspartate aminotransferase (n = 22) 226 U/L (IQR 56–449 U/L), lactate dehydrogenase (n = 18) 474 U/L (IQR 263–1,004 U/L), and prothrombin time (n = 26) 44 seconds (IQR 31–54 seconds) (Table 2).

**Table 2:** Laboratory parameters of pregnant patients from a meta-analysis of maternal and fetal outcomes of Crimean-Congo hemorrhagic fever during pregnancy

Parameter	No. patients	Median patient value (IQR)	Reference range
Leukocytes, × 10^3^ cells/mL	34	3.7 (2.8–5.1)	4–11
Hemoglobin, g/dL	17	10.0 (9.0–11.1)	12–16
Platelet count, × 10^3^/µL	35	58 (24–115)	150–400
Alanine aminotransferase, U/L	22	80 (25–190)	0–40
Aspartate aminotransferase, U/L	22	226.5 (56–448.5)	0–37
Lactate dehydrogenase, U/L	18	473.5 (262.5–1,004)	120–250
Creatinine phosphokinase, U/L	12	90 (70.8–962)	30–200
Prothrombin time, s	26	13.25 (11.4–15.0)	8.9–11.1
Activated partial thromboplastin time, s	26	43.5 (30.9–53.8)	24–35
International normalized ratio	13	1.07 (1.00–1.22)	0.8–1.2

### Outcome Analysis

Maternal outcomes were reported for 52 women, among whom 15 (28.8%) died. Fetal outcomes were reported in 54 pregnancies, 31 (57.4%) of which resulted in fetal loss. Maternal case-fatality rates (CFRs) increased with gestational age at infection: CFRs were 0 (0/7) for first trimester, 18.8% (3/16) for the second trimester, and 31.3% (5/16) for third trimester. Fetal loss was variable across trimesters. First trimester pregnancies had the highest fetal CRF, 85.7% (12 deaths in 14 cases). We noted a sharp decrease in fetal CFR for the second trimester, with a 31.3% (5/16) fatality rate. However, we observed an increasing trend in the third trimester, for which the fetal CFR was 52.9% (9/17) cases (Table 1; Figure 3).

**Figure 3:**
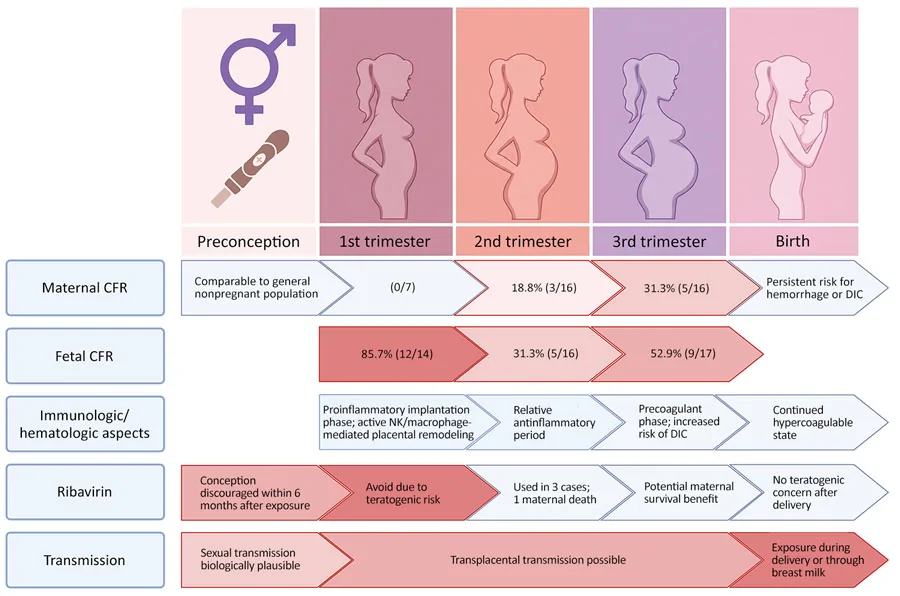
The summary of the maternal and fetal outcomes from a meta-analysis of maternal and fetal outcomes of Crimean-Congo hemorrhagic fever during pregnancy. Graphic shows transmission risk and the role of ribavirin by trimester. CFR, case-fatality rate; DIC, disseminated intravascular coagulation.

We performed a sensitivity analysis restricted to laboratory-confirmed cases, excluding 3 cases diagnosed by autopsy, 3 by clinical criteria alone, and 9 without available diagnostic information. In that restricted cohort, maternal CFR was 13.2% (5/38) and fetal CFR was 47.5% (19/40). Although both of those CFRs were lower than in the full cohort, trimester-specific patterns were similar; we observed no maternal deaths in the first trimester and persistently high fetal loss throughout pregnancy.

Ribavirin use (received or not) was reported for 43 pregnancies, among which 32.6% (14/43) patients received ribavirin. Maternal outcome was available for 33 of 43 cases with known ribavirin therapy (received or not). Only 1 (7.1%; 1/14) patient who received ribavirin died, whereas the CFR was 31% (9/29) for mothers who did not receive ribavirin (OR 0.17 [95% CI 0.02–1.51]; p = 0.128). We performed a subgroup analysis for the association between ribavirin and maternal death per trimester. No patients received ribavirin therapy during the first trimester. Among 3 patients treated with ribavirin during the second trimester, 1 (33.3% CFR) died. Of 11 other patients who received ribavirin, no deaths were reported for 6 treated during the third trimester nor for 5 whose trimester at ribavirin administration was not reported. We noted a statistically significant difference in maternal death between groups who did and did not received ribavirin in third trimester (OR 0.33 [95% CI: 0.13–0.84]; p = 0.031) (Table 3).

**Table 3:** Association of ribavirin usage with maternal death from a meta-analysis of maternal and fetal outcomes of Crimean-Congo hemorrhagic fever during pregnancy*

Cohort	Outcomes reported, no. (%)	Maternal death		p value
		No. (%)	Odds ratio (95% CI)	
Total cohort, n = 55	52 (94.5)	15 (28.8)		
Ribavirin treatment reported†	43 (82.7)		0.171 (0.019–1.513)	0.112
No ribavirin	29 (67.4)	9 (20.9)	Referent	
Ribavirin	14 (32.6)	1 (2.3)		
First trimester	6 (11.5)		NA	NA
No ribavirin	6 (100)	0		
Ribavirin	0	0		
Second trimester	16 (30.8)	3 (18.8)	2.750 (0.162–46.8)	0.489
No ribavirin	13 (81.2)	2 (66.7)	Referent	
Ribavirin	3 (18.8)	1 (33.3)		
Third trimester	14 (26.9)	5 (35.7)	0.333 (0.132–0.840)	**0.031**
No ribavirin	8 (57.1)	5 (100)	Referent	
Ribavirin	6 (42.9)	0		

*Bold font indicates statistical significance. NA, not applicable. †Includes case reports of determination of whether to administer ribavirin. Trimester at ribavirin administration was not reported for 5 patients, none of whom died.

## Discussion

In this meta-analysis integrating published and previously unpublished cases, we applied a patient-level analytical framework to examine CCHF during pregnancy. By harmonizing clinical manifestations, laboratory parameters, gestational timing, treatment exposure, and maternal and fetal outcomes at the patient level, we were able to explore trimester-specific risk patterns and treatment associations. Our findings suggest that CCHF in pregnancy is associated with substantial maternal mortality rates and high rates of fetal loss, highlighting pregnancy as a clinically relevant modifier of disease course and outcome.

The overall maternal CFR of 28.8% observed in our cohort exceeds that reported in most contemporary nonpregnant CCHF cohorts, where the fatality rate typically ranges from 5% to 20% (*7*). Of note, the patient-level approach enabled stratification by gestational age, revealing a clear increase in maternal mortality rates with advancing pregnancy. We observed no maternal deaths for first-trimester infection, whereas mortality rates rose substantially in the second and third trimesters (Table 1). That gestational gradient might reflect the cumulative physiologic burden of pregnancy, including increasing plasma volume, progressive cardiopulmonary demands, and heightened vulnerability to hemorrhage in late gestation (*14*,*15*). In contrast, fetal loss was most pronounced for first-trimester infection; >80% of CCHFV infections during early pregnancies resulted in fetal demise. That finding is biologically plausible because early gestation is characterized by rapid placental development and heightened susceptibility to systemic inflammation and maternal viremia (*16*,*17*).

In sensitivity analyses restricted to laboratory-confirmed cases, rates of both maternal death and fetal loss were lower. However, trimester-specific patterns of maternal and fetal outcomes remained similar. The observed reduction in absolute maternal mortality rates likely reflects differences in case ascertainment, including exclusion of postmortem diagnoses and cases without confirmed testing, as well as variability in clinical management and supportive care. The persistence of trimester-specific trends supports the robustness of the observed gestational risk gradient.

Consistent with prior reports (*10*), the most frequent clinical features included fever, mucosal bleeding, myalgia, and headache (Table 1). Vaginal bleeding was observed in nearly one quarter of cases, underscoring the dual obstetric and infectious risk profile of CCHF in pregnancy. Laboratory evaluation demonstrated profound thrombocytopenia, leukopenia, transaminase elevation, and coagulation abnormalities (Table 2), which are hallmarks of severe CCHF. Pregnancy-associated hemostatic changes likely amplify those imbalances, contributing to the high incidence of hemorrhagic complications observed. The association between maternal death and laboratory abnormalities could not be performed because so few cases were available.

The relatively high frequency of vaginal bleeding in our cohort demonstrates a crucial bedside challenge. In pregnant patients, hemorrhagic manifestations of CCHF can overlap with obstetric emergencies, such as placental abruption, placenta previa, miscarriage, or postpartum hemorrhage. Placental abruption is particularly relevant in the second half of pregnancy, where vaginal bleeding, abdominal pain, uterine tenderness, and fetal distress can dominate the clinical presentation (*18*). However, those findings might be difficult to distinguish from systemic viral hemorrhage in patients with CCHF, where thrombocytopenia, coagulopathy, and endothelial dysfunction are prominent features (*1*). As a result, vaginal bleeding in endemic settings should prompt consideration of both obstetric and infectious etiologies, particularly when accompanied by systemic symptoms, such as fever and myalgia.

Beyond obstetric causes of bleeding, HELLP (hemolysis, elevated liver enzymes, and low platelets) syndrome represents a key diagnostic mimic. Thrombocytopenia and elevated liver enzymes are central features of both HELLP syndrome and severe CCHF. Hemolysis can further complicate laboratory interpretation, particularly in patients receiving ribavirin, which is known to induce hemolytic anemia (*19*). In that context, distinguishing between pregnancy-specific disorders and viral hemorrhagic disease can be challenging. Therefore, in patients with thrombocytopenia, transaminase elevation, and bleeding, especially in endemic regions or with relevant exposure history, CCHF should be considered alongside HELLP syndrome and other hypertensive disorders of pregnancy (*20*). Early multidisciplinary assessment is essential because delays in recognizing either condition could adversely affect maternal and fetal outcomes.

Pregnancy is characterized by complex, stage-dependent immunologic adaptations aimed at maintaining fetal tolerance while preserving antimicrobial defense (*21*). Those changes include modulation of innate immune responses, shifts in T-cell polarization, altered interferon signaling, and dynamic cytokine regulation (*21*,*22*). Although such adaptations are physiologically essential, they could impair effective antiviral immunity and permit higher or more sustained viral replication during infections like CCHF (*23*). The placenta plays a central role in that interaction. Increasing evidence from viral hemorrhagic fevers suggests that the placenta might serve as a site of viral persistence, immune activation, and endothelial injury (*24*). Placental infection can result in local inflammation, microvascular damage, and disruption of the maternal–fetal barrier, predisposing to fetal hypoxia, intrauterine fetal demise, and preterm labor (*25*). In advanced gestation, the highly vascularized placental bed could also act as a source of ongoing viral replication and hemorrhage, potentially exacerbating maternal disease severity.

Vertical transmission of CCHF virus has been reported in several cases, but its true incidence remains unknown because of inconsistent neonatal testing and follow-up (*12*,*26*). Potential routes include transplacental transmission during maternal viremia, exposure to infected maternal blood during delivery, and postnatal contact with infectious bodily fluids. Of note, we found 4 PCR-positive and 1 IgG-positive cases reported in the literature (Table 1). In our dataset, neonatal outcomes were inconsistently documented, reflecting a critical gap in the literature and highlighting the need for standardized neonatal evaluation in future cases.

Although direct evidence for CCHFV transmission through breast milk is limited (*27*), viral RNA has been detected in blood and body fluids during acute infection, as well as in the convalescent phase (*28*). In 1 reported case, breast milk was CCHFV IgM-positive despite being PCR-negative (*29*). Therefore, temporary avoidance of breastfeeding during the viremic phase is prudent, particularly in cases of maternal bleeding or severe disease.

Ribavirin remains the most widely used antiviral agent for CCHF. Recent observational studies have suggested a potential positive role in reducing risk for death, particularly when initiated early in the disease course (*30*,*31*). However, its clinical application in obstetric populations is complicated by its classification as a category X drug. That designation stems from its potent teratogenic and embryocidal effects documented across multiple animal models, where even low-dose exposure resulted in substantial craniofacial, skeletal, and ocular malformations (*19*,*32*). In our meta-analysis, no patients infected during the first trimester received ribavirin, reflecting prevailing concerns regarding fetal toxicity and the absence of safety data in early pregnancy. Of note, ribavirin use was associated with lower observed maternal mortality rates, particularly in the third trimester, during which no deaths occurred among treated patients, and we observed a statistically significant difference in maternal deaths compared with untreated patients (Table 3). However, the number of patients who received ribavirin in the third trimester was limited. Thus, those findings must be interpreted cautiously and considered hypothesis-generating. Because treatment decisions were made across diverse clinical settings, differences in disease severity, timing of clinical manifestations, and overall clinical management might have influenced outcomes. Nevertheless, the lower maternal mortality observed among ribavirin-treated patients in later gestational periods aligns with clinical practice in several endemic regions, where maternal survival is prioritized once fetal organogenesis is complete. 

The major strength of this study lies in its patient-level design, which enabled standardized variable definitions, gestational age–specific analyses, and direct comparison of treated and untreated patients. The inclusion of unpublished cases further enhances representativeness and mitigates publication bias toward unusually severe CCHF manifestations. Limitations include the retrospective nature of included data, incomplete reporting of outcomes, and heterogeneity in diagnostic and treatment practices across regions and decades. Variations in supportive care, including transfusion strategies, management of hemorrhagic manifestations, and access to intensive care, might have contributed to outcome variability. Laboratory confirmation was unavailable for some historical cases, and detailed timing of ribavirin initiation was missing for nearly all cases.

In conclusion, CCHF during pregnancy represents a critical clinical challenge characterized by a high maternal CFR and devastating neonatal outcomes. The unique immunologic environment of pregnancy, together with the placenta’s role as a potential viral reservoir, exacerbates disease severity and creates a substantial risk for vertical transmission. Our findings suggest that although CCHFV infection during the first trimester is associated with the highest rates of fetal loss, advanced gestation carries a higher risk for maternal hemodynamic collapse and catastrophic hemorrhage. Despite its known teratogenic risks, ribavirin could be considered in selected cases as part of life-saving maternal management. The small number of laboratory-confirmed neonatal CCHFV infections and inconsistent reporting of outcomes limit our understanding; further research into the long-term outcomes of survivors is needed to address the current gaps in obstetric and pediatric CCHF care. Ultimately, the high mortality rates observed in this cohort underscore the urgent need for standardized clinical protocols and for more structured CCHF surveillance and follow-up strategies in endemic regions.

Appendix 1 Additional information on a meta-analysis of maternal and fetal outcomes of Crimean-Congo hemorrhagic fever during pregnancy.

Appendix 2 Published studies included in a meta-analysis of maternal and fetal outcomes of Crimean-Congo hemorrhagic fever during pregnancy.
