# Supplementary material for: Meta-analysis of Maternal and Fetal Outcomes of Crimean-Congo Hemorrhagic Fever during Pregnancy
Source: Emerg Infect Dis. 2026 Oct;32(10):1551–60. doi: 10.3201/eid3210.260383 (PMC13632869; doi:10.3201/eid3210.260383)
Supplement: Supplementary file 1 [file 26-0383-Techapp-s1.pdf]

Article DOI: https://doi.org/10.3201/eid3210.260383

EID cannot ensure accessibility for supplementary materials supplied by authors. Readers who have difficulty accessing supplementary content should contact the authors for assistance.

Meta-analysis of Maternal and Fetal Outcomes of Crimean-Congo Hemorrhagic Fever during Pregnancy

Appendix 1

Databases and Search Terms

Database: PubMed

Date of search: October 31, 2025

Search fields: Title/Abstract [tiab], MeSH Terms

Search string:

((“pregnancy”[MeSH] OR Gestation OR “Pregnant People”[Mesh] OR pregnan*

OR “maternal health”[MeSH] OR “maternal outcome*” OR “fetal outcome*”

OR “perinatal outcome*” OR fetal OR “fetus”[MeSH])

AND (“Hemorrhagic Fever, Crimean”[Mesh] OR “Crimean Congo Hemorrhagic Fever”

OR “CCHF” OR “Crimean Congo virus” OR “tick-borne viral hemorrhagic fever”

OR “nairovirus infection” OR “tick-borne disease” OR “Congo Virus Infection”))

Results retrieved: 41 records

Database: Ovid MEDLINE (R) and Epub Ahead of Print, In-Process, In-Data-Review & Other Non-Indexed Citations, Daily and Versions (1946–October 30, 2025)

Date of search: October 30, 2025

Search fields: .af. (All fields – Title, Abstract, Keywords)

Search string:

((Gestation or pregnan* or “maternal outcome*” or “fetal outcome*”

or “perinatal outcome*” or fetal)

and (“Crimean Congo Hemorrhagic Fever” or “CCHF” or “Crimean Congo virus”

or “tick-borne viral hemorrhagic fever” or “nairovirus infection”

or “tick-borne disease” or “Congo Virus Infection”)).af.

Results retrieved: 36 records

Database: Scopus

Date of search: October 31, 2025

Search fields: TITLE-ABS-KEY

Search string:

TITLE-ABS-KEY (Gestation OR pregnan* OR “maternal outcome*” OR “fetal outcome*”

OR “perinatal outcome*” OR fetal)

AND TITLE-ABS-KEY (“Crimean Congo Hemorrhagic Fever” OR “CCHF”

OR “Crimean Congo virus” OR “tick-borne viral hemorrhagic fever”

OR “nairovirus infection” OR “tick-borne disease” OR “Congo Virus Infection”)

Results retrieved: 161 records

Database: Web of Science Core Collection

Date of search: October 31, 2025

Search fields: Topic (TS)

Search string:

#1 TS = (Gestation OR pregnan* OR “maternal outcome*” OR “fetal outcome*”

OR “perinatal outcome*” OR fetal)

#2 TS = (“Crimean Congo Hemorrhagic Fever” OR “CCHF” OR “Crimean Congo virus”

OR “tick-borne viral hemorrhagic fever” OR “nairovirus infection”

OR “tick-borne disease” OR “Congo Virus Infection”)

#3 #2 AND #1

Results retrieved: 33 records

Database: Cochrane Library

Date of search: October 31, 2025

Search fields: Title, Abstract, Keyword

Search string:

#1 Gestation OR pregnan* OR maternal outcome* OR fetal outcome*

OR perinatal outcome* OR fetal

#2 “Crimean Congo Hemorrhagic Fever” OR “CCHF” OR “Crimean Congo virus”

OR “tick-borne viral hemorrhagic fever” OR “nairovirus infection”

OR “tick-borne disease” OR “Congo Virus Infection”

#3 #1 AND #2

Results retrieved: 1 record

Appendix Table. Summary of data search

Database	Total Retrieved	After Deduplication
PubMed	41	
Ovid MEDLINE (R)	36	
Scopus	161	
Web of Science	33	
Cochrane Library	1	
Total	272	176
